# Isoform differences drive functional diversity of NHR-49

**DOI:** 10.17912/micropub.biology.001655

**Published:** 2025-08-01

**Authors:** Lexus Tatge, Peter M. Douglas

**Affiliations:** 1 Molecular Biology, The University of Texas Southwestern Medical Center, Dallas, Texas, United States; 2 Molecular Biology, Hamon Center for Regenerative Science and Medicine, Dallas, Texas, United States

## Abstract

The *C. elegans* nuclear hormone receptor, NHR-49 , is a critical regulator of lipid metabolism, which possesses five isoforms differing predominantly within their N-termini. Yet functional distinctions between these different isoforms remain largely unexplored. Using CRISPR-based N- and C-terminal epitope tagging with the biotin ligase, TurboID, we observed that the longest isoform displays a more dynamic subcellular localization, partitioning between nucleus and cytoplasm. Proximity labeling revealed differences in interactomes with N-terminally tagged long isoform of NHR-49 enriched for cytoplasmic proteins, including endocytic machinery like RAB-10 and RAB-11.1 , while C-terminal tags associated primarily with inner nuclear pore components and transcriptional regulators. These findings highlight isoform-specific differences for NHR-49 which dramatically impact its subcellular localization and interaction networks. Our study reveals a previously uncharacterized layer of regulatory complexity in nuclear receptor biology, which emphasize the importance of isoform preferences when interpreting functional genomics data in *C. elegans* and beyond.

**Figure 1. Isoform specific localization, abundance, and interactomes of endogenous NHR-49:**
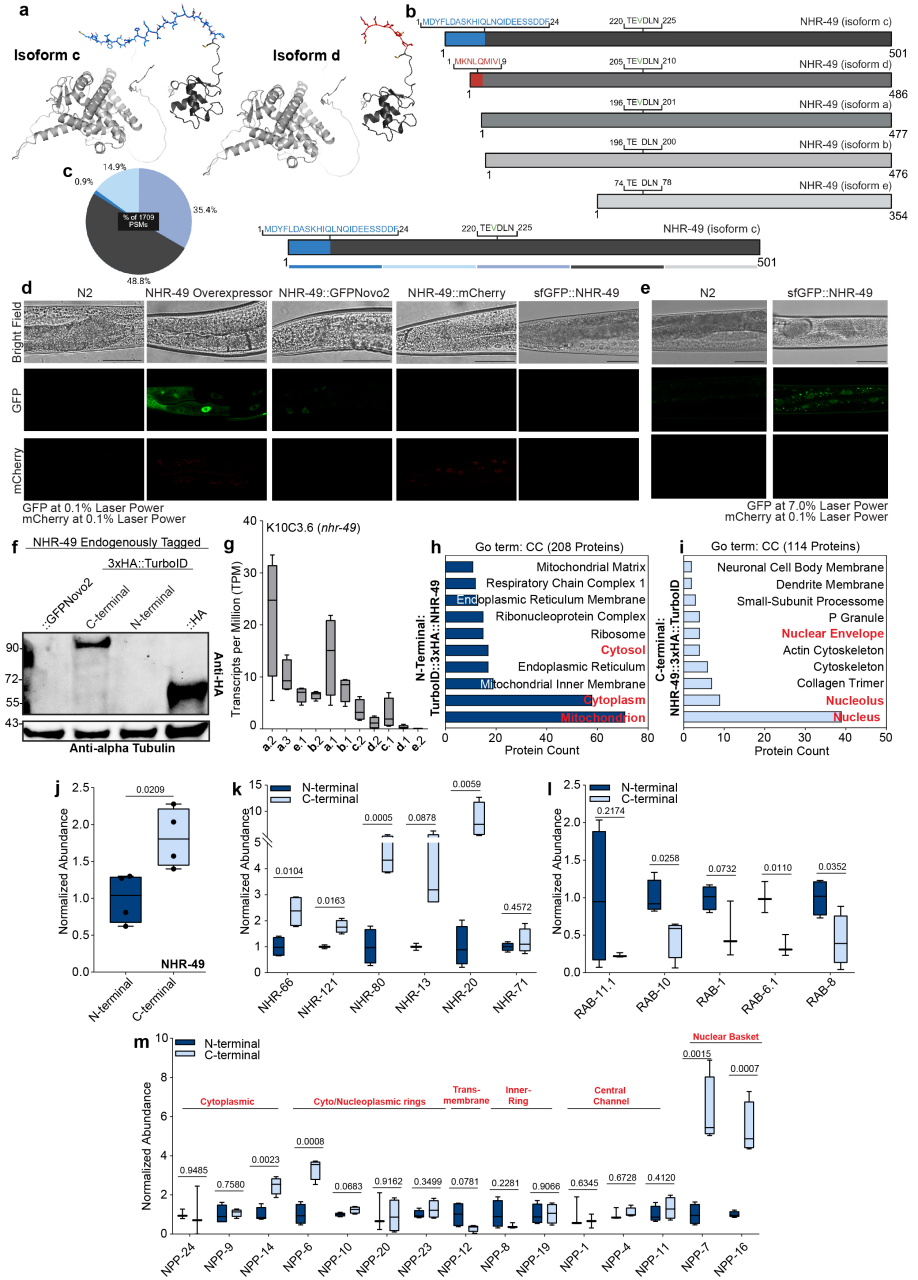
a. Structural prediction depicts N-terminal variation of NHR-49 's isoform C in blue and isoform D in red. DNA binding domain in dark grey, ligand binding domain in light grey. b. Schematic illustrates isoform variation of NHR-49 's five protein products. NHR-49 's isoform C variation in blue, isoform D variation in red, isoform C, D, and A's included Valine in green, and conserved amino acids in varying greys. c. Pie chart annotating the percent of peptide spectrum matches (PSMs) per 100 amino acids of NHR-49 across it's longest isoform. Dark blue denotes first 100 amino acids, light blue denotes second 100 amino acids, purple, dark grey, and light grey, are the 3 ^rd^ , 4 ^th^ , and 5 ^th^ 100 amino acids. d. Fluorescent micrographs of Day 1 worms of varying NHR-49 strains. N2 (non-transgenic wild type), NHR-49 ::GFP *( utsIs4 )* overexpressor, endogenous NHR-49 ::GFPNovo2 *( syb2863 )* , endogenous NHR-49 ::mCherry *( syb5674 )* , and endogenous sfGFP:: NHR-49 *( syb9651 )* , from left to right. Scale bar = 50μm. GFP laser at 0.1%, mCherry laser at 0.1%. e. Fluorescent micrographs of Day 1 worms of varying NHR-49 strains. N2 (non-transgenic wild type) and endogenous sfGFP:: NHR-49 *( syb9651 )* , from left to right. Scale bar = 50μm. GFP laser at 7.0%, mCherry laser at 0.1%. f. Western blot analysis of different endogenously tagged NHR-49 strains. NHR-49 ::GFPNovo2 as a non-HA control, NHR-49 ::3xHA::TurboID *( syb10204 )* , TurboID::3xHA:: NHR-49 *( syb10203 )* , and NHR-49 ::HA *( syb2927 )* , from left to right. Primary antibodies were monoclonal mouse HA and monoclonal rabbit alpha-tubulin. g. Box and whisker plot of NHR-49 transcripts per million (TPM) from RNA sequencing of N2 worms at Day 1 *ad libitum* . h. GO Term cellular component of proteins biotinylated only in the N-terminally tagged NHR-49 TurboID *( syb10203 )* after background N2 subtraction. i. GO Term cellular component of proteins biotinylated only in the C-terminally tagged NHR-49 TurboID *( syb10204 )* after background N2 subtraction. j. Box and whisker plot with representative data points of the normalized abundance of N- or C-terminally tagged NHR-49 TurboID biotinylating NHR-49 . Mean ± SEM. *n* = 4. Unpaired t-test used for statistics. k. Box and whisker plot with representative data points of the normalized abundance of N- or C-terminally tagged NHR-49 TurboID biotinylating other Nuclear Hormone Receptors (NHRs). Mean ± SEM. *n* = 2-4. Unpaired t-test used for statistics. l **.** Box and whisker plot with representative data points of the normalized abundance of N- or C-terminally tagged NHR-49 TurboID biotinylating Rab GTPases (RABs). Mean ± SEM. *n* = 2-4. Unpaired t-test used for statistics. m. Box and whisker plot with representative data points of the normalized abundance of N- or C-terminally tagged NHR-49 TurboID biotinylating Nuclear Pore Proteins (NPPs). Mean ± SEM. *n* = 2-4. Unpaired t-test used for statistics.

## Description

Nuclear hormone receptors (NHRs) are a highly conserved family of transcription factors that play critical roles in development, metabolism, and stress responses across metazoans (Chawla, Repa et al. 2001, Evans and Mangelsdorf 2014). Their functional diversity is traditionally attributed to their differential abilities to bind distinct ligands, recognize specific DNA sequences at Hormone Response Elements (HREs), and recruit diverse co-activators or co-repressors (Weikum, Liu et al. 2018). This modular architecture enables complex regulation of transcriptional programs in response to environmental and physiological cues (Claessens and Gewirth 2004, Kumar, Johnson et al. 2004). While these mechanisms have been extensively studied in mammals, where humans possess 48 nuclear receptors, much less is known about the functional diversity among the 284 nuclear receptors encoded in the *Caenorhabditis elegans* genome (Escriva, Delaunay et al. 2000, Owen and Zelent 2000, Maglich, Sluder et al. 2001, Sluder and Maina 2001, Escriva, Bertrand et al. 2004, Zhang, Burch et al. 2004, Sural and Hobert 2021). Even less attention has been given to potential functional differences between isoforms of individual nuclear receptors.

Arguably one of the most extensively characterized nuclear receptors in *C. elegans* , NHR-49 , mediates lipid catabolism, beta-oxidation, regulation of lifespan, immune response, and many other processes (Van Gilst, Hadjivassiliou et al. 2005, Lee, Goh et al. 2016, Hu, D'Amora et al. 2018, Doering, Ermakova et al. 2023). Based solely on sequence similarity, a clear ortholog for NHR-49 is the mammalian Hepatic Nuclear Factor 4, HNF4 gamma, which similarly activates beta-oxidation (Gerdin, Surve et al. 2006). Despite its physiological importance, the functional distinctions among NHR-49 's five endogenous isoforms remain largely unexplored. These isoforms differ primarily in their N-terminal regions, with three of the five containing an additional valine approximately 280 amino acids upstream relative to the conserved C-terminal ( **Fig. 1a, b** ). This N-terminal variation suggests that previously unrecognized mechanisms of isoform-specific functionality may contribute to the functional breadth of NHR-49 .

Detection of proteolytic fragments from ectopically overexpressed NHR-49 ::GFP *( utsIs4 )* by mass spectrometry reveals a disproportionate ratio of N- versus C-terminal peptides of the protein ( **Fig. 1c** ). With 71 peptides detectable including 15 unique peptides, and over 10,000 peptide spectrum match (PSM) coverage, the N-terminus of NHR-49 displays less than 1.0% sequence coverage at day 1 of adulthood, consistent with rapid turnover or conformational masking, while the middle 300-400 amino acids contained almost half of the fragmentation at 48.8%. This differential PSM coverage further suggests that the N-terminus may serve as an important regulatory role by influencing isoform behavior and protein-protein interactions.

Transgenic studies involving the ectopic overexpression of gene products often select the longest isoform of a gene to ensure that all potential protein domains are retained, and no functional regions are inadvertently omitted. This is further confounded when transgenes are constructed with cDNA versus the intron-containing genomic sequence, which are more likely to retain post-transcriptional processing like splicing. However, the question already remained as to whether one isoform predominates over others and whether such dominance is tissue or organellular specific (Ezkurdia, Rodriguez et al. 2015). This is particularly relevant when investigating NHR-49 , as groups have historically relied on transgenic worms overexpressing the long isoform of NHR-49 c (Van Gilst, Hadjivassiliou et al. 2005, Burkewitz, Morantte et al. 2015, Lee, Goh et al. 2016, Watterson, Tatge et al. 2022). During the course of our laboratory's research on NHR-49 , we were intrigued by mass spectrometry experiments performed on two endocytic trafficking proteins, RAB-10 and RAB-11.1 , upon RNAi knockdown of the Heat Shock Transcriptional factor 1, *hsf-1* (Watterson, Arneaud et al. 2022). Among the complex mixture of interacting proteins, NHR-49 emerged as the only detected nuclear hormone receptor, out of the 284 different NHRs in the worm, which displayed differential association under knockdown of *hsf-1* (Watterson, Arneaud et al. 2022).

To expand upon these findings, we utilized available transgenic worms overexpressing NHR-49 ::GFP from its own all-tissue promoter as an extrachromosomal array (Ratnappan, Amrit et al. 2014). To ensure stable and consistent expression of NHR-49 ::GFP for further examination, we integrated the transgene into the genome via irradiation, followed by five rounds of outcrossing (Watterson, Tatge et al. 2022). Analysis of these transgenic worms revealed that NHR-49 ::GFP bound a number of cytosolic proteins, including several involved in endocytic trafficking. These observations were consistent with our prior experiments which identified endogenous NHR-49 in complex with ectopically expressed GFP:: RAB-11.1 and GFP:: RAB-10 (Watterson, Arneaud et al. 2022, Watterson, Tatge et al. 2022). Yet, these reciprocal experiments hinge on ectopic overexpression of different gene products.

With the advent of widespread CRISPR-based genome editing in *C. elegans* , we and others engineered epitope tags at the endogenous *nhr-49* locus. To our knowledge, all available endogenous reporters insert epitope tags at the C-terminus of *nhr-49* , encompassing all isoforms. These endogenously tagged strains exhibit substantially lower steady state levels as predicted but also exhibit strong nuclear localization without cytosolic signal above the typical background fluorescence in worms. While these observations seemingly contrast those reported for the NHR-49 ::GFP overexpressor, low fluorescence signal from the endogenous protein combined with high autofluorescence in the cytosol confound results and by no means rule out its cytosolic localization (Watterson, Tatge et al. 2022). Upon correspondence with other laboratories, we constructed a set of endogenously tagged *nhr-49* strains including a C-terminal NHR-49 ::mCherry ( *syb5674 )* as well as an N-terminal sfGFP:: NHR-49 *( syb9651 )* , which is specific for the long isoform c. In parallel, the laboratory of Dr. Stefan Taubert generously provided their NHR-49 ::GFPNovo2 ( *syb2863 )* . In contrast to the NHR-49 ::GFP *( utsIs4 )* overexpression construct, which displays a variable nucleocytoplasmic fluorescence distribution, these endogenously tagged strains exhibit low overall fluorescence with nuclear enrichment that was marginally distinguishable from non-transgenic autofluorescence ( **Fig. 1d** ). This discrepancy raises questions as to whether the cytoplasmic localization of the transgenic NHR-49 ::GFP *( utsIs4 )* overexpressor was not physiological relevant but rather the result of its overexpression. Alternatively, could subcellular discrepancies result from isoform-specificity and the overexpression of less abundant isoforms?

To probe further, we compared non-transgenic N2 animals to those endogenously expressing the N-terminal sfGFP:: NHR-49 *( syb9651 )* protein, increasing laser power to 7.0% to enhance detectability. At lower laser power (0.1% in the GFP channel), fluorescence is undetectable. With the laser set to 7.0%, a clear nuclear signal of NHR-49 is observed. Notably, however, we also detect increased fluorescence in the cytoplasm, exceeding the autofluorescence levels of non-transgenic animals ( **Fig. 1e** ). While these findings do not negate the nuclear localization of NHR-49 , they raise the question as to whether NHR-49 exists in the cytosol and high intestinal autofluorescence masks this distribution?

To address this, we opt to take a biochemical approach rather than rely on fluorescence visualization. To this end, we generated additional *C. elegans* strains with epitope tags inserted at the endogenous *nhr-49* locus: one with an N-terminal TurboID::3xHA:: NHR-49 *( syb10203 )* fusion and another with a C-terminal NHR-49 ::3xHA::TurboID *( syb10204 )* fusion. To assess relative protein abundance, we performed HA-based detection on Day 1 adult lysates from both strains. Notably, the N-terminal NHR-49 *( syb10203 )* tagged protein is undetectable by western blot, while the C-terminal *( syb10204 )* tag showed expression at day 1 of adulthood ( **Fig. 1f** ). Next, we leveraged the TurboID tag and proximity labeling techniques to define the NHR-49 interactome. Since there was detection of the isoform C mRNA reads, yet weak detection of steady-state protein levels, we hypothesize that the long isoform of NHR-49 is transcriptionally reduced or rapidly degraded by Day 1 of adulthood ( **Fig. 1g** ). For this reason, proximity labeling serves an important role and will enable labeling of interacting proteins throughout development. This cumulative biotinylation might reveal meaningful insights into its dynamic localization and transient protein interactions (Sanchez and Feldman 2021).

Using immunoprecipitation of biotinylated proteins with streptavidin followed by mass spectrometry (LC-MS/MS), we compared Day 1 non-transgenic N2 worms with Day 1 worms expressing either the N- or C-terminally tagged NHR-49 . Accounting for the endogenously biotinylated proteins using the N2 control, we identify a shared set of 1,349 proteins biotinylated in both TurboID-tagged strains. Additionally, 208 proteins are uniquely biotinylated in the N-terminally tagged strain, while 114 are uniquely enriched in the C-terminal condition ( **Fig. 1h,i** ). Gene Ontology (GO) analysis via DAVID (Huang da, Sherman et al. 2009, Sherman, Hao et al. 2022) reveals distinct subcellular localization patterns: proteins tagged by the N-terminal TurboID are predominantly associated with the mitochondrion, cytoplasm, and cytosol ( **Fig. 1h** ), whereas those tagged by the C-terminal TurboID are enriched for nuclear, nucleolar, and nuclear envelope components ( **Fig. 1i** ). In the N-terminal strain, we do not believe that the mitochondrial labeled proteins results from non-specific biotinylation due to the fact that 49.5% of the N-terminally tagged proteins are also found enriched in immunoprecipitations of NHR-49 ::GFP. Despite a core set of shared interactions, divergence between the N- and C-terminus is highly suggestive that isoform differences can influence subcellular distribution and regulatory dynamics of the receptor.

Close examination of the interaction networks between the N- and C-terminal NHR-49 reveals insightful clues regarding its subcellular regulation. First, we observe comparable levels of NHR-49 biotinylation from both the N- and C-terminal transgenes, indicating potential homodimerization ( **Fig. 1j** ). Consistent with others, both NHR-49 strains labeled a number of established nuclear receptor binding partners via heterodimerization such as NHR-66 , NHR-121 , NHR-80 , NHR-13 , NHR-20 , and NHR-71 , however these are significantly enriched in the C-terminally tagged strain (Pathare, Lin et al. 2012, Reece-Hoyes, Pons et al. 2013, Ratnappan, D. et al. 2016) ( **Fig. 1k** ). Notably, NHR-46 and NHR-32 are detected exclusively in the C-terminal samples. In contrast, no nuclear receptors are uniquely labeled in samples containing only the N-terminal long isoform of NHR-49 . These observations raise the possibility that isoform C may function as a monomer or homodimer, whereas the shorter isoforms might engage in heterodimerization. Alternatively, isoform C may localize preferentially to the cytosol under *ad libitum* conditions, limiting its interaction with nuclear-localized partners.

In support of this hypothesis, we observe that the N-terminal tag preferentially labels several Rab GTPases on the endocytic vesicles such as RAB-11.1 , RAB-10 , RAB-1 , RAB-6.1 , and RAB-8 ( **Fig. 1l** ). Interestingly, both the N- and C-terminal tagged proteins label several nuclear pore proteins to the same degree. However, nuclear pore basket proteins facing the nucleoplasm such as NPP-7 , NPP-16 and NPP-21 (only found in the C-terminal sample), are highly enriched in the C-terminus over N-terminus. These findings indicate that the N-terminally tagged NHR-49 preferentially labels cytoplasmic proteins including endocytic vesicles when compared to the C-terminal fusion protein. However, it still retains the capacity to label some nuclear localized proteins, also observed more prominently in the C-terminal fusion protein. In contrast, the C-terminally tagged NHR-49 preferentially labels inner nuclear basket components potentially opening up a new line of investigation regarding the transcriptional dynamics underlying NHR-49 docking and activity on the nuclear pore complex (Ge, Brickner et al. 2025).

These findings highlight that tagging strategy and isoform context profoundly influence NHR-49 's abundance, localization, and interaction networks. While the C-terminal tag reinforces its canonical nuclear role through interactions with known transcriptional partners, the N-terminal tag bolsters results obtained from the overexpressed NHR-49 ::GFP strain demonstrating its connection to endocytic vesicles in the cytosol. This duality further indicates the dynamic nucleocytoplasmic distribution of NHR-49 and highlights isoform-specific contributions to these subcellular fluctuations.

Together, our results reveal that NHR-49 isoforms exhibit unexpected complexity in subcellular localization, abundance, and protein interaction profiles, driven in part by differences at the N-terminus and by the position of tagging. The striking divergence in proximity labeling of proteins between N- and C-terminally tagged strains suggests that specific isoforms of NHR-49 may participate in distinct biological processes, ranging from nuclear transcriptional regulation to vesicular trafficking. These findings challenge the assumption that all isoforms behave similarly and call for a more nuanced, isoform-resolved approach to nuclear receptor biology in *C. elegans* .

To support this shift, we offer this work as a resource to the *C. elegans* community. All newly generated NHR-49 strains described here will be made available through the CGC. Furthermore, we are providing the proximity labeling datasets, enabling researchers to query potential interactions with proteins of interest. We hope this work serves as both a technical reference and a launch point for future exploration of NHR-49 isoform-specific biology.

## Methods

**Strain Maintenance and Growth Conditions**

*Caenorhabditis elegans* strains were propagated on nematode growth medium (NGM) plates seeded with Escherichia coli OP50 and maintained at 15°C. For experimental use, animals were chunked and expanded at 20°C. All assays were conducted on NGM plates containing carbenicillin and IPTG to enable both antibiotic selection and RNAi induction. Prior to use, these plates were seeded with *E. coli* HT115 carrying the empty vector RNAi construct. To generate synchronized populations, gravid adults were subjected to alkaline hypochlorite treatment, and their eggs were collected, plated, and cultured until either the L4 larval stage or Day 1 of adulthood, as specified in the figure legend. A complete list of the strains used in this study is provided in the Reagents section.

For collection purposes for confocal, western blotting, and mass spectrometry, *Caenorhabditis elegans* were harvested at specific age-related stages, as indicated in the corresponding figure legends. Animals were washed from culture plates using M9 buffer and transferred into 15 mL conical tubes. After two additional M9 washes, the worm pellets were moved to 1.5 mL microcentrifuge tubes for downstream applications.

**Biorender Schematic**

Schematics presented in this publication were created using BioRender. Isoform-specific information for NHR-49 was obtained from publicly available datasets and tools on WormBase ( www.wormbase.org ) and Uniprot ( https://www.uniprot.org/uniprotkb/ ).

**In silico modeling of NHR-49**

The predicted structure of NHR-49 isoform C was obtained from the AlphaFold Protein Structure Database ( https://alphafold.ebi.ac.uk/entry/O45666 ) (Jumper, Evans et al. 2021) and visualized using PyMOL (v. 3.1). Since a predicted structure for isoform D is not available on AlphaFold, we employed PyMOL's mutagenesis wizard to introduce the sequence changes necessary to approximate the structural configuration of isoform D based on isoform C.

**Confocal**

After the initial washes mentioned in strain maintenance and growth conditions, the supernatant was carefully removed, and 50 µL of 200 mM levamisole was added to induce paralysis. Immediately following paralysis, 27 µL of the worm suspension was mounted on microscope coverslips and sealed with clear nail polish.

Confocal imaging was performed using a Leica SP8 confocal microscope, equipped with a photomultiplier tube, two high-sensitivity HyD hybrid detectors, and a suite of stable laser lines (UV/405 nm DMOD compact, 488 nm, 552 nm, 638 nm). Imaging utilized Leica PL APO CS2 objectives (10×/0.40 NA air, and 40×/1.30 NA oil). N2 worms were first used to adjust GFP and mCherry signal to due to the worm's natural intestinal auto-fluorescence. Once adjusted to the lowest signal possible to minimize false-positive signals, we proceeded with the fluorescent strains. Image analysis was conducted with LAS X software (v. 3.5.5).

**Preparation of Worm Extracts for Western blot and Proteomics**

Following the initial washing steps described in the Strain Maintenance and Growth Conditions section, worm pellets were transferred to 1.5 mL microcentrifuge tubes with minimal residual buffer. To prepare whole-worm extracts, samples were subjected to bead beating using a mixture of glass and zirconia beads in a non-native lysis buffer composed of 100 mM HEPES (pH 7.4), 300 mM NaCl, 2 mM EDTA, 2% Triton X-100, 1% SDS, and an EDTA-free protease inhibitor cocktail (Roche). Lysates were clarified by centrifugation at 4,000 × *g* for 5 minutes at 4°C for western blotting applications, or at 400 × *g* for 5 minutes at 4°C when preparing samples for proteomic analysis. Total protein concentrations were determined using the Pierce BCA Protein Assay Kit (Thermo Scientific, Cat. # PI23225), and all lysates were normalized to equal concentrations using the same lysis buffer.

**Western Blot**

Normalized protein samples, prepared as described in the *Preparation of Worm Extracts for Western Blot and Proteomics* section, were separated by SDS-PAGE on bis-acrylamide gels and transferred onto nitrocellulose membranes for immunoblotting. Protein bands were detected and visualized using Image Lab software (v. 6.1.0, build 7; Bio-Rad Laboratories, 2020). Primary antibodies were monoclonal mouse HA at 1:1,333 (Invitrogen - #26183) and monoclonal rabbit alpha-tubulin at 1:5,000 (Sigma - SAB5600206). Secondary antibodies were IRDye 680RD anti-rabbit (VWR - 102673-410) and 800CW anti- mouse IgG (VWR - 102673-328) both at 1:10,000 concentrations.

**RNA-seq Data Source**

RNA sequencing data analyzed in this study were previously generated as part of a broader investigation examining gene expression changes during a particular stress. The full dataset, including differential gene expression and pathway-level analyses, is currently under review for publication (Tatge et al., in revision). Here, we focus on exon-level read mapping and transcript isoform resolution, which has not been previously reported, utilizing Qiagen Bioinformatics CLC Workbench (v9.5).

**Immunoprecipitation and Mass Spectrometry**

Immunoprecipitation of biotinylated proteins using streptavidin-coated beads was performed based on the (Sanchez and Feldman 2021), with minor modifications. All lysates were prepared in non-native lysis buffer, and subsequent washes on Day 2 were also carried out using the same buffer. Clarification was achieved by centrifugation at 400 × *g* for 5 minutes at 4°C. To optimize binding efficiency, bead volume was adjusted to maintain an approximate protein-to-bead ratio of 8 µg/µL; for example, 50 µL of beads were used for 400 µL of lysate normalized to 1 mg/mL. Finally, bead-bound samples were submitted to the UT-Southwestern Mass Spectrometry Core Facility in a “dry” format, meaning all buffer was removed, and only beads were transferred into 1.5 mL microcentrifuge tubes for processing.

Once with mass spectrometry core, the magnetic bead-bound proteins were covered with 80 µL of 2 M urea in 100 mM Tris-HCl (pH ~8.0). Samples were reduced with tris(2-carboxyethyl)phosphine hydrochloride (TCEP; Sigma-Aldrich), alkylated with iodoacetamide (Sigma-Aldrich), and subsequently digested overnight with trypsin (Pierce). Following digestion, peptides were purified using an Oasis HLB µElution plate (Waters) via solid-phase extraction. Eluted peptides were reconstituted in 11 µL of 2% (v/v) acetonitrile (ACN) and 0.1% trifluoroacetic acid (TFA) in water.

For LC-MS/MS analysis, 5 µL of each sample was injected onto a Q Exactive HF mass spectrometer (Thermo Scientific) interfaced with an Ultimate 3000 RSLC-Nano liquid chromatography system. Peptides were loaded onto a 75 µm inner diameter, 15-cm EasySpray analytical column (Thermo Scientific) and separated using a 90-minute linear gradient from 0% to 28% buffer B. Buffer A consisted of 2% ACN and 0.1% formic acid in water, while buffer B contained 80% ACN, 10% trifluoroethanol, and 0.1% formic acid in water.

The mass spectrometer was operated in positive ion mode with a spray voltage of 2.5 kV and an ion transfer tube temperature of 300 °C. Full MS scans were acquired in the Orbitrap at a resolution of 120,000. Data-dependent MS/MS acquisition was performed in the ion trap, targeting the top 20 most intense ions per full scan with charge states from 2+ to 8+ using higher-energy collisional dissociation (HCD). Dynamic exclusion was set to 20 seconds.

Raw MS files were processed using *Proteome Discoverer* v. 3.0 (Thermo Scientific). Peptide identification was performed using the Sequest HT algorithm against the *C. elegans* reviewed protein database from UniProt (downloaded May 5, 2022; 26,537 entries). Search parameters included a precursor mass tolerance of 10 ppm, fragment mass tolerance of 0.02 Da, and up to three missed tryptic cleavages. Carbamidomethylation of cysteine was set as a fixed modification, while methionine oxidation was included as a variable modification. Peptide-spectrum matches were filtered using a false discovery rate (FDR) threshold of 1%. Protein quantification was based on the summed intensities of all matching peptides.

**Analysis**

All statistical analyses were performed using GraphPad Prism (v. 10.1.0). Detailed information regarding statistical approaches, including sample sizes ( *n* ), precision metrics (such as SEM or 95% confidence intervals), statistical tests applied, and significance criteria, can be found in the figure legends or directly within the figures. A p-value of less than 0.05 was considered statistically significant.

To ensure high data quality and minimize false positives in the biotinylated mass spectrometry, a stringent multi-step normalization and filtering process was applied to protein abundance data.

Step 1: Within-Condition Normalization

For each condition ( N2 T1–T4; N-terminal T1-T4; C-terminal T1-T4), total protein abundances were summed across replicates and averaged. Each protein value within a replicate was then normalized by dividing by a condition-specific scaling factor derived as:

*Normalization Factor = Sum of the Abundances _Replicate_ / Average of Sums across T1 – T4*

Zero or missing values were imputed with 1000 to avoid loss during log or ratio calculations.

Step 2: Protein Quality Filtering by Presence

Proteins were annotated as *KEEP* or *REMOVE* based on their presence across replicates. A protein was marked *REMOVE* for a condition if more than two of its replicates contained only imputed values (i.e., 1000). Otherwise, it was marked *KEEP* .

Step 3: Cross-Condition Filtering Proteins were discarded if labeled *REMOVE* in all three experimental conditions ( N2 , N-terminal, and C-terminal). Additionally, proteins marked *KEEP* only in N2 but *REMOVE* in both N- and C-terminal datasets were excluded.

Step 4: Background Subtraction and Second Filtering To account for background signals, the average abundance in N2 was subtracted from the N- and C-terminal conditions:

*Corrected Abundance = Condition Abundance – N2 Average*

Negative values resulting from subtraction were replaced with 0. Proteins were again annotated as *KEEP* or *REMOVE* if two or more replicate values were zero.

Step 5: Final Protein Filtering

Proteins labeled *REMOVE* in both N- and C-terminal conditions were excluded from further analysis.

Step 6: Data Stratification and Enrichment Analysis

The remaining data were stratified into three categories: Proteins uniquely present in the N-terminal dataset, proteins uniquely present in the C-terminal dataset, and proteins common to both, for which enrichment ratios and statistical comparisons (e.g., fold change and p-values) were computed.

## Reagents

**Table:** 

**Strain Name**	**Genotype and Notes**	**Publication and Availability**
N2	Wildtype worm strain	CGC
PMD166	*utsIs4* [ *nhr-49* p:: NHR-49 ::GFP; *myo-3* p::mCherry ( *nr2041* +)]	Parental strain is PMD150 (available on CGC), and before that, the non-integrated from (Ratnappan, Amrit et al. 2014).
PMD342	*nhr-49* ( *syb5674* [ NHR-49 ::mCherry] I)	Designed with SunyBiotech for this manuscript. SunyBiotech Source Name: PHX5674
PMD300	*nhr-49* c( *syb9651* [sfGFP:: NHR-49 c])	Designed with SunyBiotech for this manuscript. SunyBiotech Source Name: PHX9651
STA08	*nhr-49* ( *syb2927* [ NHR-49 ::HA])	From the laboratory of Dr. Stefan Taubert. SunyBiotech Source Name: PHX2927
STA07	*nhr-49* ( *syb2863* [ NHR-49 ::GFPNovo2])	From the laboratory of Dr. Stefan Taubert. SunyBiotech Source Name: PHX2863
PMD319	*nhr-49* ( *syb10204* [ NHR-49 ::3xHA::TurboID]) GGCG linker included between tag and endo NHR-49 .	Designed with SunyBiotech for this manuscript. SunyBiotech Source Name: PHX10204
PMD320	*nhr-49* c( *syb10203* [3xHA::TurboID:: NHR-49 c]) GGCG linker included between tag and endo NHR-49 .	Designed with SunyBiotech for this manuscript. SunyBiotech Source Name: PHX10203

## Data Availability

Description: Proteomics Data. Resource Type: Dataset. DOI: https://doi.org/10.22002/9at93-n9r73
